# The effect of substance P on asthmatic rat airway smooth muscle cell proliferation, migration, and cytoplasmic calcium concentration *in vitro*

**DOI:** 10.1186/1476-9255-8-18

**Published:** 2011-07-21

**Authors:** Miao Li, Yun-Xiao Shang, Bing Wei, Yun-Gang Yang

**Affiliations:** 1 Department of Pediatrics, No.2 Hospital of China Medical University, Shenyang 110004, China

## Abstract

Airway remodeling and airway hyper-responsiveness are prominent features of asthma. Neurogenic inflammation participates in the development of asthma. Neurokinin substance P acts by binding to neurokinin-1 receptor (NK-1R). Airway smooth muscle cells (ASMC) are important effector cells in asthma. Increases in ASMC proliferation, migration, and cytoplasmic Ca^2+ ^concentration are critical to airway remodeling and hyper-responsiveness. The effects of substance P on ASMC were investigated in Wistar rats challenged with a previously described asthmatic rat model. To exclude possible influences from other factors, the role of substance P was also investigated in primary cultured rat ASMC. Substance P and WIN62577-induced changes in cytoplasmic Ca^2+ ^concentration were observed by fluorescence microscopy, and expression of Ca^2+ ^homeostasis-regulating genes was assessed with real-time PCR. We found that cytoplasmic Ca^2+ ^concentration increased in normal rat ASMC treated with substance P, but decreased in asthmatic rat ASMC treated with WIN62577, an antagonist of NK-1R. Real-time PCR analysis revealed increased *Serca2 *mRNA expression but decreased *Ip3r *mRNA expression after WIN62577 treatment in asthmatic rat ASMC. Flow cytometric analysis (FCM) revealed that most asthmatic rat ASMC stayed at G_1 _phase after combined treatment with WIN62577 and IL-13 *in vitro*. Transwell analysis suggested that ASMC migration was reduced after WIN62577 treatment. Therefore, we conclude that NK-1R is related to asthma mechanisms and a NK-1R antagonist downregulates calcium concentration in asthmatic ASMC by increasing *Serca2 *mRNA and decreasing *Ip3r *mRNA expression. The NK-1R antagonist WIN62577 inhibited ASMC IL-13-induced proliferation and ASMC migration *in vitro *and therefore may be a new therapeutic option in asthma.

## Introduction

Asthma is a chronic inflammatory disease of the lower airways associated with various comorbidities and characterized by variable, often reversible, airway obstruction [1]. Airway hyper-responsiveness is a hallmark of asthma and seems to be related to chronic airway inflammation [2]. Thus, anti-inflammatory treatment with inhaled corticosteroids is the cornerstone of pharmacotherapy for persistent asthma [1]. However, corticosteroids do not fully suppress asthma-associated airway inflammation, particularly for asthma airway remodeling; therefore many new therapeutic options to control airway inflammation are being explored.

In asthmatic airways, ASMC proliferate and migrate, especially during airway remodeling [3]. ASMCs are not only important effector cells but also inflammatory cells in asthma. The responsiveness of smooth muscle to diverse stimuli is controlled by changing the concentration of intracellar calium ion ([Ca^2+^]_i_). Elevation of [Ca^2+^]_i _results from increased Ca^2+ ^influx across the plasma membrane following activation of Ca^2+^-permeable ion channels and the Na^+^-Ca^2+^-exchanger (NCX, 3Na^+^:1Ca^2+^), and from release of stored Ca^2+ ^from the sarcoplasmic reticulum (SR) triggered by inositol 1,4,5-triphosphate receptor (IP3R) or ryanodine receptor (RyR) channels [4]. Impaired replenishment of SR stores arising from reduced activity of the sarco/endoplasmic reticulum Ca^2+ ^(SERCA) pump result in increased Ca^2+ ^concentration, which can in turn impact a wide range of Ca^2+^-dependent smooth muscle functions [5]. Abnormal Ca^2+ ^handling by ASMC has been proposed previously to be an important determinant of the airway hyper-responsiveness that is characteristically present in asthma [6,7]. Mahn K *et al*. reported a deficiency of SERCA in asthmatic patients as compared to healthy control subjects [8]. Therefore, drugs able to inhibit ASMC proliferation and migration or to decrease ASMC calcium concentration may be beneficial in alleviating airway hyper-responsiveness.

Tachykinins such as substance P and neurokinin A belong to a family of peptides that are released from airway nerves after noxious stimulation [9]. Tachykinins have been proposed to play an important role in human respiratory diseases such as bronchial asthma and chronic obstructive pulmonary diseases (COPD), as they have been shown to activate the neurokinin (NK)-1 and NK-2 receptors, leading to potent effects on airway smooth muscle tone and secretions, bronchial circulation, and inflammatory and immune cells [10]. Tachykinin levels were increased in induced sputum from asthmatic and cough patients with acid reflux [11]. Furthermore, in contrast to non-asthmatic control subjects, increased NK-1 and NK-2 receptor mRNA expression had been demonstrated in the airways of asthma patients [12]. However, the role of neurokinins in the asthmatic airway and ASMC is unknown. Therefore, in the present study, we investigated the effect of substance P on the asthmatic airway in an asthmatic rat model and cultured ASMC with the aim of identifying new methods to alleviate airway hyper-responsiveness and remodeling.

## Methods and materials

### Asthmatic rat model

Thirty healthy female Wistar rats weighing 150-160 g were purchased from the experimental animal center of China Medical University. All experimental protocols involving animals were approved by the China Medical University Animal Care Committee and complied with the guidelines of the China Council on Animal Care. The animals were randomly divided into two groups of 15. Asthmatic rats were prepared according to previously described methods using a modified ovalbumin (OVA) (Sigma-Aldrich, Beijing, China.) immunization protocol developed to induce allergic asthma in rats [13]. Briefly, subcutaneous injection of 1 mg OVA and 200 mg/ml aluminum hydroxide (Sigma-Aldrich, Beijing, China) in 1 ml PBS and intraperitoneal (ip) injection of 1 ml heat-killed Bordetella pertussis bacteria (6 × 10^9^/ml, Beijing, China) were administered on day 0 and day 7. Rats in the control group were treated with 1 ml PBS containing only 200 mg/ml aluminum hydroxide. Two weeks later, the rats were placed in a transparent glass chamber (approximately 20 cm × 20 cm × 20 cm in volume) connected to an ultrasonic nebulizer (model 100, Yadu, Shanghai, China) and subjected to repeated bronchial allergen challenge by inhalation of OVA (2%) for 20 min/day for 6 days. Rats in the control group were challenged with PBS.

### Bronchial responsiveness to methacholine

To investigate OVA-induced effects on airway responsiveness, we measured respiratory parameters induced by methacholine (MCh). After the rats were challenged, they were anesthetized with pentobarbital (30 mg/kg ip). The trachea was cannulated with a 14-gauge tube. The rats were quasisinusoidally ventilated with a computer-controlled small-animal ventilator (flexiVent; SCIREQ, Montreal, Quebec, Canada) with a tidal volume of 8 ml/kg set automatically depending on body weight, at 90 breaths/min and positive end-expiratory pressure of 3.0 cmH_2_O. Airway resistance was measured by the forced oscillation technique. 5 doses of MCh (Sigma-Aldrich, Beijing, China) solution (10-160 μg/ml) in 0.5 ml PBS every 1 min. MCh was delivered via jugular veins intermittently by intravenous injection. After each methacholine challenge, the respiratory system resistance was recorded by computer animal pulmonary function analysis software testing baseline airway resistance and Re, which represents changes in airway responsiveness. When Re reached or exceeded the baseline Re 2 times stop to push Mch.

### Bronchoalveolar lavage (BAL) and cell counting

After the measurement of lung responsiveness, the rats were disconnected from the ventilator and killed with an overdose of pentobarbital. A catheter was then inserted into the trachea, and BAL was performed. The cell suspension was concentrated by centrifugation (1000 rpm, 10 min. at 4°C), and the cell pellet was resuspended in 1 ml saline. To perform the differential leukocyte cell count, 0.1 ml of the cell suspension was drop on a glass slide and stained with Wright-Giemsa stain. A microscope was then used to examine 400 nucleated cells.

### IgE level in plasma

Twenty-four hours after the last challenge, rats were anaesthetized with pentobarbital, and blood was collected from the heart. Plasma total IgE measurement was performed using rat IgE ELISA quantification kit (R&D ELISA KIT, DoBio Biotech, Shanghai, China).

### Hematoxylin and eosin staining

Routine histological staining methods were applied. The middle lobe of the right lung sections of 5-μm were stained with hematoxylin and eosin (HE) for general histological evaluation.

### Airway smooth muscle cell culture

Primary ASMC were cultured according to a previously described method [14]. Tracheas were dissected, excised, and washed aseptically. The tracheal internal and external membrane layers were removed. The smooth muscles were separated longitudinally from cartilage and digested in 0.1% trypsin, 0.02% EDTA, and 0.2% type IV collagenase for 30 min in a shaking water bath at 37°C. The harvested cells were collected and cultured with DMEM-F-12 medium (1:1 vol/vol) (Thermo Scientific HyClone, Beijing, China) supplemented with 10% FBS (Thermo Scientific HyClone, Beijing, China). The medium was changed every 3-4 days. When the ASMC were confluent and elongated spindle shape, and grew with the typical hill-and-valley appearance, the cells were passaged with 0.25% trypsin-0.02% EDTA solution. Three passages were performed, every 10-14 days. At the fourth passage, ASMC were used for experiments. ASMC were identified with anti α- actin (1:200 diluted in PBS, Boster Biotechnology, Wuhan, China) and FITC-conjugated goat-anti-rabbit (1:100, Invitrogen, Beijing, China) and observed with a fluorescence microscope.

### Ca^2+ ^concentration measurement

The cells were divided randomly into 3 groups: control group, substance P-induced, and WIN62577-induced group. Cells in the WIN62577-induced group were treated with 10^-8 ^M NK-1R antagonist WIN62577 (Sigma-Aldrich Co, Beijing, China); those in the substance P-induced group were treated with 10^-5 ^M substance P (Sigma-Aldrich Co, Beijing, China). After washing with PBS, the ASMC were dropped onto glass coverslips (≈1 × 10^3 ^cells/coverslip) and incubated for 30 min at 37°C with 5 μM Fura-2 AM (F-1221, Eugene Oregon, USA), a radiometric Ca^2+ ^indicator, for loading. They were then observed under a fluorescence microscope (IX70, Olympus, Japan) combined with a double-excitation microfluorimeter. The light emitted by the cells at 510 nm during excitation at wavelengths of 340 and 380 nm was recorded. The ratio of the intensities of emission (R_340/380_) was taken as a measure of [Ca^2+^]_i_. For each image, regions of interest were defined within single cells, and the average fluorescence intensity of each region of interest was measured.

### Real-time PCR analysis

To investigate the expression of genes involved in Ca^2+ ^storage at the SR, real-time PCR was performed for quantitative analysis of *Serca2 *(*Atp2a2*) and *Ip3r *mRNA expression in different group. After collection of primary cultured cells from control and asthma-induced rats. The cells come from asthmatic rats were divided into 2 groups: untreatment and WIN62577-treatment group. Cells in the WIN62577-treatment group were treated with 10^-8 ^M NK-1R antagonist WIN62577 (Sigma-Aldrich Co, Beijing, China) for 24 h; those in the untreatment group were treated with PBS. Total RNA was extracted from ASMC using RNAiso™ Plus reagent (Takara, Dalian, China) and quantified using a spectrophotometer. Following quantification, 2 μg RNA was reversely transcribed to cDNA, and real-time quantitative PCR assays were conducted using an ABI PRISM 7500 real-time PCR System (Applied Biosystems, Foster City, CA, USA). PCR amplification was performed using the SYBR PrimeScript™ RT-PCR kit reagent (Takara, Dalian, China). The PCR conditions for SERCA2 and IP3R were 45 cycles of denaturation at 95°C for 5 s, annealing and extension at 60°C for 30 s. For quantification, a standard curve was generated with various dilutions of the cDNA templates. Target mRNA levels were normalized to those of GAPDH. The following oligonucleotide primers were used: *Serca2 *forward 5'-GAAGCAGTTCATCCGCTACCTCA-3', reverse 5'-GCAGACCATCCGTCACCAGA-3'; *Ip3r *forward 5'-CAGGAACGTGGGCCATAACA-3', reverse 5'-TCCAGAGCTTCATCGCCATC-3'. Gene expression was analyzed by the 2^-ΔΔ*CT *^method.

### Detection of ASMC proliferation

The role of WIN62577 on ASMC proliferation induced by IL-13 was next investigated. After ASMC from control rats were digested with 0.25% trypsin and counted, cells were seeded (8,000 cells/well) into 3 parallel wells and divided into different intervention groups (PBS, IL-13, and WIN62577 with IL-13) for 24 h, 48 h and 72 h. IL-13 (10^-5 ^M, Sigma-Aldrich Co.) and WIN62577 (10^-8 ^M) were added to medium when cells were seeded. MTT (5 mg/ml, Sigma-Aldrich Co.) was added 4 h before detection. After incubation, 200 μl DMSO was added to each well, the plate was shaken gently for 10 min at room temperature, and absorbance was obtained at 490 nm using a microplate reader to generate an absorbance growth curve.

To study the effect of WIN62577 on the ASMC cell cycle, FCM was used. After purified ASMC collected from control rats were treated with different interventions (PBS, 10^-5 ^M IL-13, and 10^-8 ^M WIN62577 with IL-13) for 24 h, the cells were collected, washed with PBS, and then suspended in 70% ethanol at 4°C overnight. Cells were incubated with 20 μl 0.1% RNase A for 15 min at room temperature and then incubated with 50 μg/ml propidium iodide (PI) for 15 min. Cell cycle analysis was performed using CellQuest software (Becton Dickinson, USA).

### Transwell analysis

To study the role of WIN62577 on asthmatic ASMC migration, transwell analysis was conducted after cells were harvested with trypsin and resuspended (8.0 × 10^5 ^cells/ml) in serum-free growth medium. ASMC derived from asthmatic rats were divided into 2 groups (control and intervention) and each was added to the upper chamber. For the intervention group, WIN62577 (10^-8 ^M) with 10% bovine serum albumin BSA was added to the lower chamber. The control group was induced by PBS instead. After 24 h incubation at 37°C, the membranes were removed, the cells on the upper side were scraped off, and the cells that migrated to the lower side of the membrane were fixed with 4% polyoxymethylene. The number of cells was counted in 5 random fields under 40 × magnification, and the mean was calculated.

### Statistical analysis

All experiments were repeated in triplicate. All data were expressed as mean ± SD and analysed with SPSS 17. Comparisons for 2 groups were made using Student's T-test. One-way analysis of variance (ANOVA) with SNK or LSD test was used for experiments in which more than 2 groups were compared. *P *< 0.05 was considered to be statistically significant.

## Results

### Airway responsiveness to MCh

**Figure 1:**
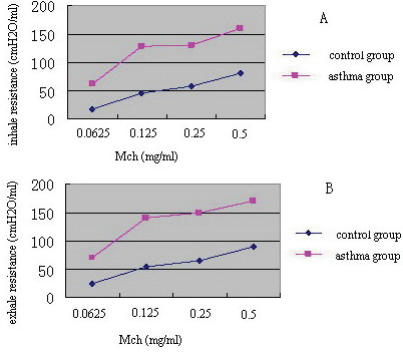
**Airway responsiveness to MCh**. Asthmatic rat inhale resistance and exhale resistance increased when compared with normal rat. A: representive inhale resistance; B: representive exhale resistance.

To test the airway responsiveness of asthmatic rats *in vivo*, we measured respiratory parameters induced by MCh. Airway responsiveness of rats in the asthmatic group increased in comparison to the control group after induction by MCh (Figure 1).

### Inflammatory cells in BAL fluid

The number of inflammatory cells in BAL fluid was measured and compared between OVA-sensitized and control rats. Remarkably, the total cell number in BAL fluid recovered from OVA-sensitized/challenged rats was significantly higher than that from PBS-treated rats. Total cells and eosinophils in asthmatic BAL fluid significantly increased compared with control rat's, the difference significant (*P *< 0.05); Total cells and eosinophils in the treatment group significantly decreased when compared with asthmatic group, the difference significant (*P *< 0.05), but did not significantly differ from the control group (*P >*0.05) (Table 1).

**Table 1:** Inflammation cells in different group rat's BALF ( ± s) ×10^4 ^/mL

	Total	eosinocyto	lymphocyto	granulocyto	macrophage
Asthmatic group	610 ± 32*	461 ± 31*	40 ± 16*	20 ± 6.3*	88 ± 15*
Budesonide	372 ± 13^#▲^	147 ± 23^#▲^	19 ± 3.5^#▲^	18 ± 3^#▲^	56 ± 10^#▲^
treatment group					
normal group	172 ± 21	21 ± 7.5	8.2 ± 5.0	0.0 ± 0.0	70 ± 13

*P < 0.05, compare with normal group; ^#^*P *< 0.05, compare with asthmatic group; ^▲^*P *> 0.05, compare with normal group.

### IgE measurement

Plasma total IgE was statistically significantly higher in OVA-sensitized rats compared with controls (330.6 ± 97.7 ng/ml vs 282.2 ± 22.7 ng/ml, respectively; *P *< 0.01).

### Ca^2+ ^concentration variations in asthmatic rat ASMC induced by WIN62577

**Figure 2:**
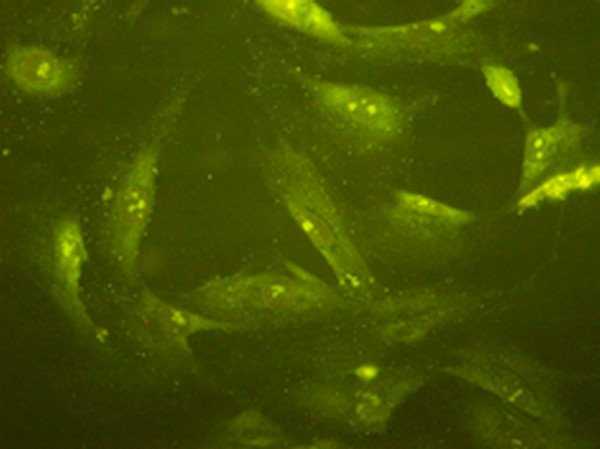
**Immunofluorescence against α-actin suggests that the green staining cell is ASMC**.

**Figure 3:**
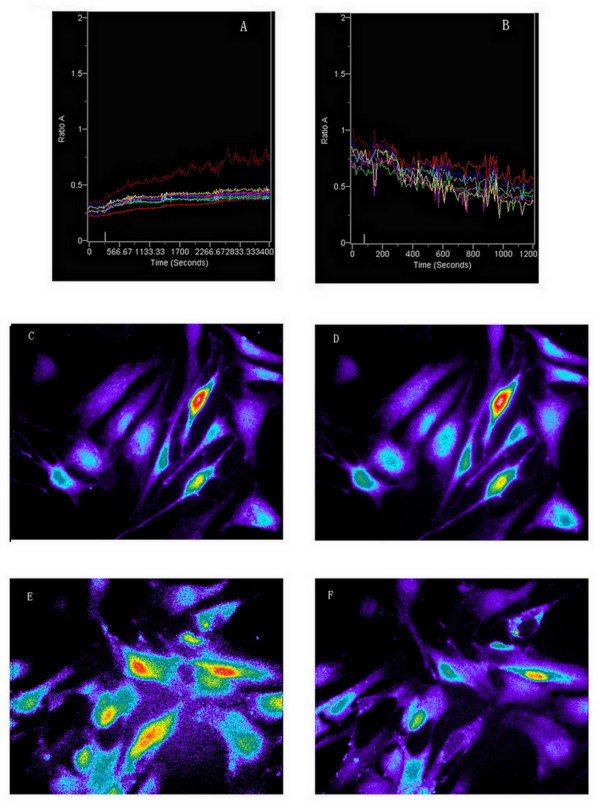
**Effects of WIN62577 on intracellular Ca^2+ ^concentration ([Ca^2+^]_i_)**. The ratio of the intensities of emission (R_340/380_) in control group was 0.2(Fig.3A), but in asthma group the ratio is 1.25, which suggest that the calcium concentration in asthma group was higher than the one in control group (Fig.3B); C and D: representive the cell induced before and after substance P. calcium concentration in ASMC was increasing intervened by substance P, the calcium concentration of asthmatic rat ASMC was decreasing intervened by substance P receptor antagonist. E and F: representive the cell induced before and after WIN62577.

The purity of ASMC was confirmed to exceed 95% by α-actin staining (Figure 2). ASMC were loaded with the Ca^2+ ^indicator Fura-2 and recorded using fluorescence microscopy. Substance P (10^-5 ^M) induced [Ca^2+^]_i _to increase in control ASMC (Figure 3A, n = 5, *P *< 0.05). In contrast, [Ca^2+^]_i _decreased in asthmatic rat ASMC exposed to WIN62577 (10^-8 ^M) (Figure 3B, n = 5, *P *< 0.05). R_340/380 _in control ASMC was 0.2, but in asthmatic rat ASMC the ratio was 1.25, suggesting that calcium concentration was higher in asthmatic ASMC than in control cells. After substance P treatment, the R_340/380 _in control rat ASMC increased to 0.5; after WIN62577 treatment, R_340/380 _decreased to 0.4 in asthmatic rat ASMC. These findings indicate that substance P had the effect of elevating calcium concentration in ASMC, while WIN62577 caused it to decline.

### *Serca2 *and *Ip3r *mRNA expression in different groups

**Figure 4:**
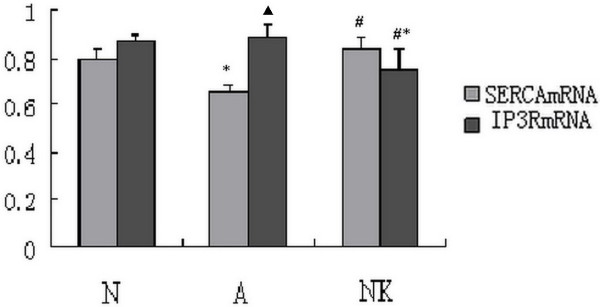
***Serca2 *mRNA and *Ip3r *mRNA express in different group**. *Serca2 *mRNA in asthmatic rat ASMC decreased compared with normal ASMC (*P *< 0.05). But after induced by WIN62577 the expression of *Serca2 *mRNA increased (*P *< 0.05). In asthmatic ASMC, we found that *Ip3r *mRNA had no difference compared with normal ASMC (*P *> 0.05). In contrast, the expression of *Ip3r *mRNA decreased after induced by WIN62577 in asthmatic ASMC (*P *< 0.05). *P < 0.05, control vs normal group; ^#^*P *< 0.05, control vs asthmatic group; ^▲^*P *> 0.05, control vs normal group. N: representive ASMC from control group; A: representive asthmatic ASMC group untreated by WIN62577; NK: representive asthmatic ASMC after WIN62577 treated.

The equilibrium of Ca^2+ ^content in the SR is maintained by SERCA pumping calcium in, while IP3R and RyR release calcium out. SERCA and IP3R are key regulators of Ca^2+ ^content in asthmatic ASMC. SERCA2 is the predominant SERCA isoform in smooth muscle. We found that *Serca2 *mRNA decreased in asthmatic ASMC compared with normal ASMC. However, after induction by WIN62577, the expression of *Serca2 *mRNA in asthmatic ASMC increased. IP3R is an SR Ca^2+ ^release channel that opens upon the binding of IP3. In asthmatic ASMC, the expression of *Ip3r *mRNA did not differ from that of control ASMC. In contrast, the expression of *Ip3r *mRNA decreased in asthmatic ASMC after induction by WIN62577 (Figure 4)

### The role of WIN62577 on ASMC proliferation and migration

**Figure 5:**
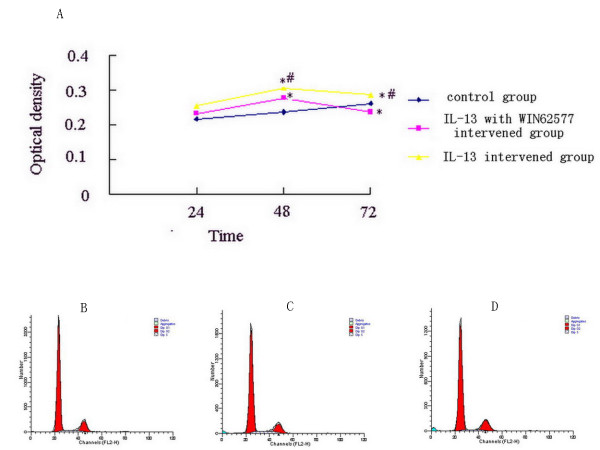
**MTT and FCM analysis the effect of NK-1R antagonist to ASMC proliferation**. In IL-13 intervention group ASMC proliferate more faster than in normal group, from 24 h to 48 h the difference become significant (*P *< 0.05). In NK-1R antagonist intervention group ASMC proliferate faster than in normal group but slower than in IL-13 intervention group, especially during 48 h to 72 h (Fig.5A). Flow cytometric analysis of ASMC cell cycle (Fig.5B, 5C, 5D). Most of ASMC stayed at G_1 _stage in WIN62577 intervention group compared with in normal group. B, C and D: representative examples of normal group, IL-13 intervention group and IL-13 with WIN62577 intervention group.

**Figure 6:**
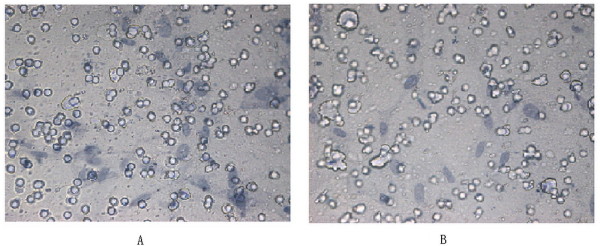
**Transwell analyzed the effect of NK-1R antagonist on ASMC migration**. The number of ASMC in WIN62577 intervened group compared with normal group the difference was statistically significant (*P *< 0.05). A and B: representive examples of normal group and WIN62577 induced group.

Because IL-13 promotes ASMC proliferation, in our study ASMC from control rats were found to proliferate faster after induction with IL-13, the differences among different groups in 48 and 72 h were statistically significant (Figure 5A, P < 0.05). Most ASMC treated with WIN62577 and IL-13 stayed at G_1 _phase compared with those induced by IL-13 alone, with a statistically significant difference between groups (Figure 5B, C, D, P < 0.05). The number of migrated cells significantly decreased after WIN62577 intervention compared with untreated control cells (P < 0.05) (Figure 6, Table 2).

**Table 2:** Transwell detect the role of WIN62577 to ASMC migration

group	Mean ± SD	n
Normal control group	23 ± 3	15
WIN62577 intervened group	16 ± 2*	15

*P < 0.05, control vs normal group.

## Discussion

Airway hyper-responsiveness and remodeling are important characteristics of asthma, and both are related to calcium levels in ASMC. In asthma, inflammatory cells can release cytokines that in turn induce increased calcium concentration in ASMC, airway smooth muscle contraction, and airway hyper-responsiveness. For example, IL-8 has been shown to increase ASMC calcium concentration [15]. Elevation of [Ca^2+^]_i _can be caused by Ca^2+ ^release from intracellular Ca^2+ ^stores or Ca^2+ ^influx from the extracellular space. ASMC plasma membrane ion channels also contribute to changes in Ca^2+ ^concentration. Over a long term, increased Ca^2+ ^concentration induces ASMC to proliferate as well as produce and secrete pro-inflammatory factors [16].

Recently Mahn *et al*. reported that a SERCA2 deficiency in ASMC contributed to their secretory and hyperproliferative phenotype in asthma, suggesting that SERCA2 may play a key role in mechanisms of airway remodeling [12]. In our study, using an asthmatic rat model we observed that Ca^2+ ^homeostasis changed in asthmatic ASMC, with increased calcium content in asthmatic rat ASMC compared to control rat ASMC. Furthermore, substance P increased the calcium concentration of control ASMC, and WIN62577 decreased the calcium concentration of asthmatic ASMC via increased expression of *Serca2 *mRNA. However, WIN62577 decreased the expression of *Ip3r *mRNA in asthmatic ASMC had no difference compared with normal ASMC. Based on these findings, we conclude that WIN62577 plays a role in decreasing calcium concentration, which may ultimately alleviate airway inflammation and responsiveness. As a result, substance P antagonist WIN62577 may be an attractive target for therapeutic approaches to asthma. Regrettably, we were unable to examine the role of WIN62577 in a variety of TRP channels, stretch-activated channels, voltage-gated channels, and Ca^2+^-dependent K^+ ^channels, although they were involved in increased calcium ion concentration.

Airway remodeling is an important characteristic of asthma. The airway pathological features of asthma include reshaping of smooth muscle cell proliferation, hypertrophy, airway epithelium metaplasia, fibrosis, increased mucous cells and blood vessels, and interstitial remodeling [17]. ASMC are very important effector cells in asthma that proliferate, migrate, and contract due to a variety of cytokines and inflammatory mediators, especially in asthma airway remodeling.

IL-13 is an important Th2 lymphocyte proinflammatory factor [18,19] that also plays an important role in chronic airway disease. IL-13 can change the integrity of the airway and increase airway sensitivity [20]. Leigh *et al*. demonstrated that the probability of airway hyper-responsiveness and remodeling decreased in IL-13 knockout mice, suggesting that IL-13 played an important role in airway remodeling [21]. IL-13 can increase the smooth muscle cell volume and change the contractile properties of smooth muscle cells and airway reactivity [22-24], as well as to promote ASMC proliferation and participate in airway remodeling [25]. Therefore, IL-13 was adopted in our experiment to induce ASMC proliferation.

MTT and FCM analysis demonstrated that WIN62577 inhibited the ASMC proliferation induced by IL-13. FCM analysis of the ASMC cell cycle suggested that most ASMC remained at G_1 _phase after WIN62577 treatment. G_1 _phase is the key to the entire cell cycle, and the cell cycle protein D is the key protein in G_1 _phase that determines transformation from G_1 _to S phase. Therefore the role of WIN62577 on protein D and other control genes should be studied further. In addition, IL-13 binds the IL-13 receptor on the cell surface to activate cell receptor protein tyrosine kinase (PTK). NK-1R is a G-protein receptor that activates the phosphatidyl inositol bisphosphate (PIP2) second messenger system to promote IP3 binding to IP3R and calcium release from the SR. The increased concentration of calcium ions could cause membrane polarization and activate the PTK to achieve its biological function [26]. However, the mechanism of how NK-1R antagonists act on the IL-13 receptor remains unknown. Therefore, the relationship between WIN62577 and IL-13 receptor should be investigated in the future.

In asthma, eosinophils, mast cells, and other cells secrete cytokines and inflammatory mediators that promote the development of asthma. Jonsson *et al*. demonstrated that substance P induced eosinophils from asthmatic patients to become active and demonstrate chemotropism [27]. In this experiment, we demonstrated that NK-1R antagonist WIN62577 had the effect of inhibiting ASMC migration *in vitro*, indicating that WIN62577 may contribute to the inhibition of airway remodeling. Taken together, our results suggest that NK-1R antagonist WIN62577 could decrease ASMC calcium concentration and inhibit ASMC proliferation and migration, and therefore may be useful to alleviate asthma airway remodeling and airway hyper-responsiveness.

## Declaration of competing interests

The authors declare that they have no competing interests.

## Authors' contributions

ML carried out the ASMC culture and participated in the Ca^2+ ^concentration detecting and drafted the manuscript YY carried out the immunoassays and ELISA detecting. YS participated in the design of the study and performed the statistical analysis. BW participated in ASMC proliferation and migration analysis. All authors read and approved the final manuscript.
